# Combined Results of Two Cross-Sectional Surveys on the Participation in Clinical Trials and the e-Consent Procedure in the Landscape of Haematology

**DOI:** 10.3390/clinpract13060133

**Published:** 2023-11-23

**Authors:** Bert Heyrman, Stef Meers, Ann Van De Velde, Sébastien Anguille

**Affiliations:** 1 Ziekenhuis Netwerk Antwerpen, Department of Haematology, 2020 Antwerp, Belgium; 2 Algemeen Ziekenhuis KLINA, Department of Haematology, 2930 Brasschaat, Belgium; 3 Department of Haematology, University Hospital Antwerp, 2650 Edegem, Belgium

**Keywords:** clinical trials, clinical trial barriers, clinical trial enrolment, patient-reported outcome, physician-reported outcome, e-consent

## Abstract

Despite the motivation of oncology patients to take part in clinical trials, only a minority of them are enrolled in clinical trials. Implementation of new practical procedures can become a barrier that withholds patients from participating in clinical trials. Treating physicians are crucial in augmenting trial accrual. The drivers that promote physicians to allocate patients for clinical trials need further assessment. We conducted two separate cross-sectional surveys, addressing patients with a haematological disease in one survey and haematologists in another survey. The patient survey was filled out by 420 patients. Significant relationships between the willingness to participate in a trial and trial knowledge (*p* < 0.001) and between doctor–patient relationship and participation willingness (*p* = 0.007) were noted. Patients above 60 years were less willing to use an electronic consent procedure vs. patients younger than 60 (*p* < 0.001). The physician questionnaire was completed by 42 participants of whom most (83%) were active in and (94%) motivated for clinical trials. Apart from the patient benefit and scientific interest, prestige was an equal motivator closely followed by financial remunerations. First goal was not to harm the patient. Our study confirms the high willingness of patients for trial participation and the need to rethink the structure of trial organisation. The e-consent procedure is not the method preferred by most patients above 60 years old.

## 1. Introduction

Randomized clinical trials provide the highest level of evidence on which a therapeutic strategy can be based [1]. Numerous treatments have failed and are failing to demonstrate safety and efficacy in large, randomized trials [2]. This confirms the absolute necessity of large trials, which implies a medical and social need to include patients whenever possible. Failure to reach a certain population has shown to negatively impact survival improvement [3]. Clinical trials are complex, expensive, and time-consuming for all involved parties [4]. To successfully complete a trial, a strong collaboration is needed between the government, private stakeholders, healthcare professionals, and patients [5]. Any reduction in the engagement of one of the parties can be detrimental for a trial. This will not only result in a loss of time and resources but more importantly a failure to improve disease-related outcome. The leading cause of premature trial closure is an insufficient accrual rate [6]. Since many decades, a minority of cancer patients are treated in the context of a clinical trial [7]. The design of the trial is the first barrier for inclusion. Due to restrictive eligibility criteria, certain populations are excluded from trial participation [8]. As a result, real-life data have gained importance in recent years [9]. Analysis of real-world data showed the potential to augment the eligible population and make trial data more representative [10]. Artificial intelligence has recently confirmed that the population of eligible patients can be doubled by making adaptations in inclusion criteria without a negative impact on patient safety [11]. Patient enrolment is performed at the hospital level where the patient and the treating physician can affect accrual. In general, patients express a high willingness for trial participation [12]. Patients are motivated by the chance to obtain new medication, by the opportunity to contribute to medical progress, and by the physician who desires for the patient to participate [13]. During the recent COVID-19 pandemic, a significant drop was seen in cancer control and prevention (CCP) trials. Enrolment to treatment trials was hardly affected, confirming the continuous need for interventional trials [14]. Patients can be hindered to participate due to protocol-related, patient-related, physician-related, or practical barriers [15]. A recent trend is the implementation of a digital informed consent (e-consent) procedure [16]. During the COVID pandemic, a remote e-consent procedure has been shown to be critical for the continuation of certain trials [17]. The e-consent procedure is well studied on practical and ethical levels, considering the perspectives of different stakeholders [18]. No information can be found on the patients’ perspectives on the implementation of an e-consent procedure.

The lack of physicians’ engagement is an important barrier for clinical trial inclusion [19]. Other physician issues can be seen as an expression of inadequate physician–patient communication (e.g., the difficulty to initiate clinical trial discussions during patient consultation) [20]. This comes on top of the possible logistical constraints [21]. Hierarchical relationships, ambition, and ego as obstacles for optimal trial inclusion are only mentioned briefly [19].

## 2. Materials and Methods

We conducted two simultaneous cross-sectional anonymous surveys in Belgium. One was addressed at patients with a haematological disease (Supplementary Material File S1), the other one addressed haematologists (Supplementary Material File S2). Our objectives were to hear the patients’ voices on the current evolution of e-consent implementation and understand the motivational factors of both patients and haematologists. Since there is no standardized survey available in this research domain, a survey was created by the authors focussing on various aspects: general information (age, gender), trial-knowledge, trial confidence, and practical aspects (Additional File S1). All questions were multiple choice to minimise the effort required for filling out the survey. The ethical committee of the University Hospital Antwerp approved the survey. During a fixed period of 6 months, patients were invited to four hospitals in the province of Antwerp, Belgium, to fill out the patient survey. All hospitals are perceived as city hospitals. Patients were invited to take part via flyers. The flyer had a QR-code that led to an online survey. Flyers were handed out by accredited haematologists. Patients were also offered the possibility to fill out the survey on-site on tablets.

The second survey addressed haematologists that were active at patient consultations. The physician survey was created by the authors with specific questions that mainly covered motivational factors for trial participation and questions on the current organisation of chimeric antibody receptor T-cell (CAR-T) therapy in Belgium. Haematologists were invited via email. An invitational email was sent to all Belgian Haematologists via the Belgian Haematology Society. Both the patient and physician surveys were only filled out online, and no data copying was performed.

Qualtrics^®^, which is licenced by the University of Antwerp, was used to create both surveys and collect all the data. Data were summarized as numbers and percentages. In the analysis of the patient survey, associations between categorical variables were assessed with chi-squared test. For the association between ordinal variables (age, doctor–patient relation) and the willingness to participate a non-parametric Mann–Whitney U test was used. All analyses were performed in IBM SPSS Statistics version 27. The interpretation of the physician survey was performed using descriptive statistical analysis. Questions related to the practical organization of CAR-T cell therapy were left out during this analysis.

## 3. Results

### 3.1. Patient Survey

#### 3.1.1. Patients

**Figure 1:**
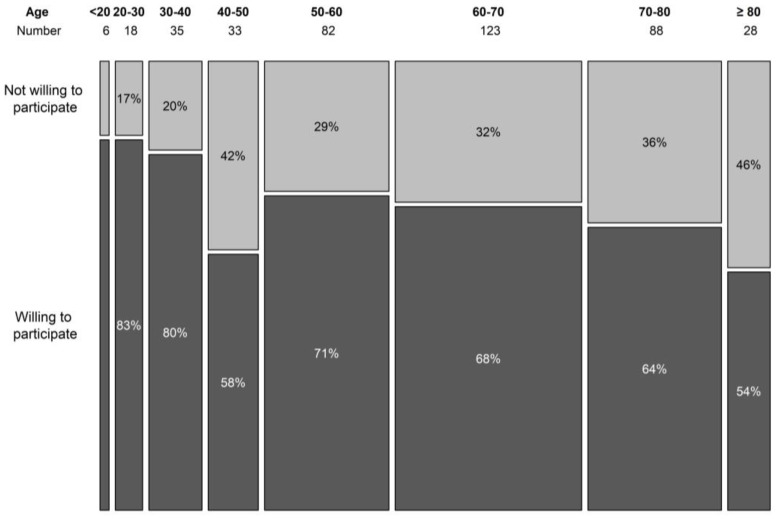
Willingness to participate in a clinical trial per age category.

During the 6-month period, 420 patients filled out the survey. The population was well balanced with 219 (52.1%) female and 187 (44.5%) male participants, and 14 (0.03%) participants did not answer the question revealing their gender. Age was registered to eight categories of 10 years (youngest <20 years, oldest >80 years). The three most represented age groups were 50–60 years, 60–70 years, and 70–80 years, containing 83, 123, and 88 patients, respectively (19.8%, 29.3%, and 21%). More than half (57%, *n* = 219) of the patients was older than 60. The willingness to participate in a trial seemed to decline with age, although the association was borderline non-significant (*p* = 0.064) (Figure 1). In the surveyed population, 86.7% indicated that they use the internet on a regular basis, and only 13.3% indicated that they hardly ever use or never use the internet. About a quarter of the patients (23.9%, *n* = 100) had taken part in a clinical trial in the past or were currently participating in a clinical trial.

#### 3.1.2. Trial Knowledge

**Figure 2:**
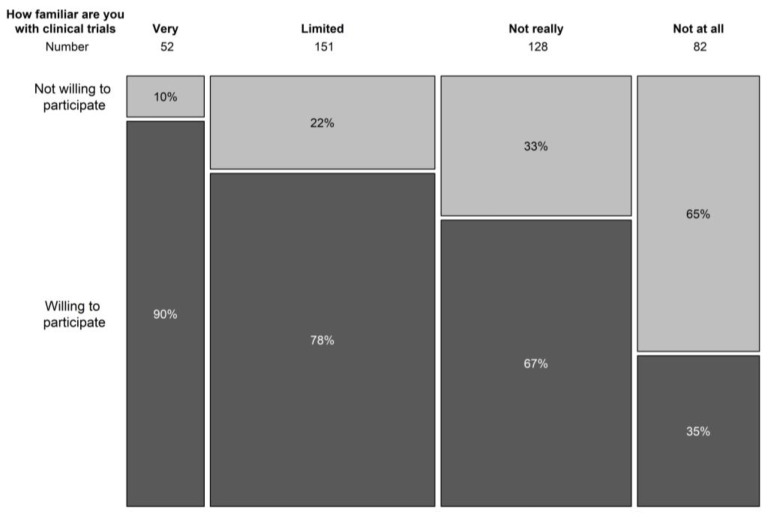
Relation between knowledge on clinical trial concepts and willingness to participate in a trial.

In terms of understanding the general concepts of clinical trials, only 12.8% indicated to be familiar with trial concepts, 37.5% had a limited knowledge of trial concepts, and 30.9% and 19.8% was not very or not at all aware of clinical trial concepts. Knowledge on the concepts of clinical trials correlated significantly with the willingness to participate in a clinical trial (*p* < 0.001) (Figure 2). In the population that never took part in a clinical trial before, 77% had never spoken with someone that had participated in a clinical trial. 

In the case of receiving an invitation to a clinical trial, patients would turn to their treating haematologist (39.9%) and general practitioner (26.5%) for more information. Other information channels were the internet (12.5%), other healthcare workers (e.g., nurses and physiotherapist) (9.4%), friends and family (4.4%), patient groups (4.0%), and social media (1.5%). A very small portion (1.75%) did not know where to gain more information from. The proportion of patients that wished to know the results of the trial they participated in was 79.9% and the ones that indicated they wished to know the results in advance were 7.6%. A small part (6.7%) was not interested in the results or were only interested in case the trail outcome would have an impact on their future treatment (5.7%).

In the population that participated in a clinical trial or was currently participating, 94.6% preferred to know the results following the trial closure. In this population, 58.1% understood all the information that was provided before trial participation or most of it (32.3%). Small number of patients (4.3%) could not understand the offered information, and 5.4% could not remember the information. The amount of information was perceived as correct by 73.4%, and 13.8% preferred more information. An equal number (both 6.4%) perceived the information to be too detailed or could not remember this information from the past. There was enough time allotted for answering questions as per 84.96% patients but not enough time was allocated as per 10.8% patients, while 4.3% could not remember the amount of time allotted. Most patients were completely (55.9%) or mostly (36.6%) satisfied with the answers provided to their questions, and only 3.2% were not satisfied. Some patients (2.15%) said they did not pose any questions or could not remember if they did (2.15%). The current format of the ICF was appreciated by 54.1% as correct, and it did not influence their decision to participate. For some patients (11.2%), the format was too scientific, and others (11.2%) found it unattractive to read. A large proportion of patients (23.5%) had no opinion on the format. 

#### 3.1.3. Trial Confidence

**Figure 3:**
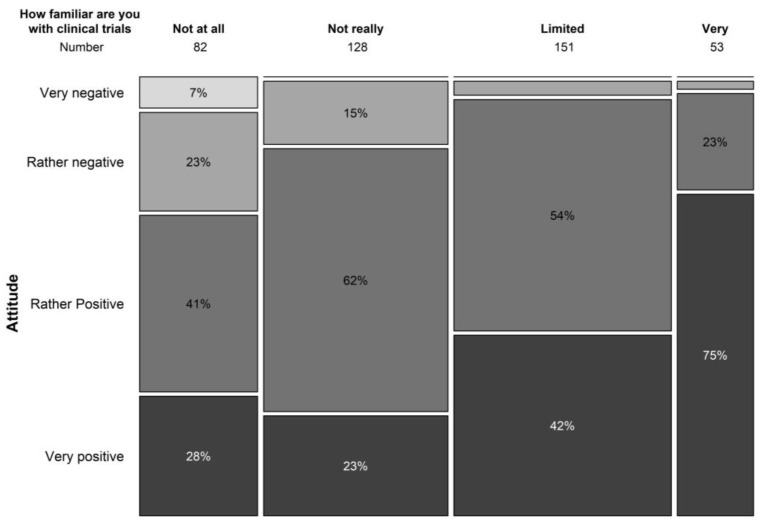
Relation between familiarity with trial concepts and attitude towards clinical trials.

The general attitude towards clinical trials was very positive in 37.9% and positive in 50% of our study population. Limited knowledge on the concepts of clinical trials did not hold back the majority of patients from having a general positive attitude towards clinical trials (Figure 3). Only 10.6% had a negative or very negative (1.5%) impression towards clinical trials. Similar results were seen in terms of safety perception with 21.3% perceiving clinical trials as extremely safe and 66.5% perceiving them as safe. In our study population, 11.1% perceived trials as not really safe and 1.7% perceived them as not safe at all. Safety concerns of clinical trials (multiple answers were allowed) were associated with the possible side effects of the treatment under investigation (40.8%), the possibility of being treated with a placebo (26.0%), and risks to the general health of patients (20.1%).

General advantages of clinical trials were mainly that they were perceived as scientific progression (43.8%), saving lives of patients (28.6%), and the amelioration of the healthcare system (25.8%). Their possible personal advantages were predominantly their aid in treating a disease (40.2%) and the satisfaction of helping others (24.6%). Other possible advantages were obtaining more time and attention from the treating physician (10%), obtaining free medication (9.3%), and aiding the family in understanding the disease (7.5%). Only 3.4% indicated that being paid for participation could be a personal advantage, and 5.1% indicated that they did not see any possible personal advantage obtained from trial participation.

The majority of patients were very willing (28%) or willing (39.7%) to take part in a clinical trial in case they would have the opportunity, and only 16.0% and 4.6%, respectively, were not really willing or not willing at all to participate in a clinical trial. About one out of ten patients (11.6%) did not know what they would do when a trial was offered to them.

**Figure 4:**
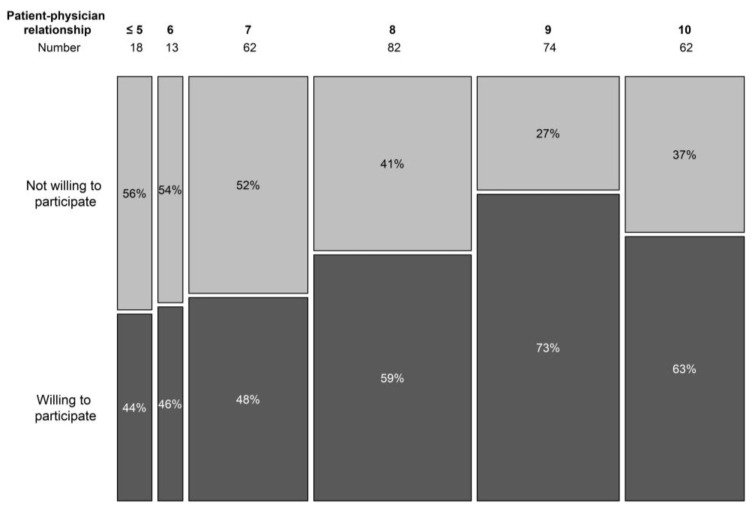
Willingness to participate in a trial per doctor–patient relationship score.

The doctor–patient relationship was scored from 1 to 10, in analogy with the visual analogue scale, used to score pain perception. A higher score expressed a better relationship. In the non-trial population, only 10% scored their relationship with the treating haematologist as 6 or below (7 in 19.9% cases, 8 in 26.4%, 9 in 23.8%, and 10 in 19.9%). In the trial population, higher scores were noted (7 or below was the score in 8.8% cases, 8 in 30.8%, 9 in 23.1%, and 10 in 37.4%). Overall, a significant correlation was seen in the doctor–patient relationship and the willingness to participate in a trial (*p* = 0.007) (Figure 4).

Overall, 75.3% of earlier trial participators would recommend study participation to others, 4.3% would recommend it only to younger patients, and 4.3% would recommend it only to the elderly. Only 2.2% would not recommend participating in a clinical trial when possible, and the rest had no opinion on this. These numbers are also reflected in the question on whether earlier participators would participate in another trial when offered one; half of the patients (51.6%) answered yes, without any condition, and 40.9% answered they would participate only if they were sure to receive a better treatment; only 5.4% answered they would participate in case there was no other alternative, and 2.2% answered they would not participate in a clinical trial again. A drawback of study participation can be the feeling of being treated as a test object. This feeling was perceived by 5.4% of earlier study participators. Another 14.0% of participators had perceived this feeling during the trial but were able to talk about it with their treating physician. Most patients (76.3%) did not have this feeling or could not remember it (4.3%).

Only six participants had refused study participation in the past. Three of them because of a lack of faith in the pharmaceutical industry, one because of the paucity of information, one because of unsatisfying answers that were given by the treating physician, and one because participation involved more frequent hospital visits in comparison to the standard of care treatment.

#### 3.1.4. Practical Aspects

Practical aspects may cause barriers in trial participation. Patients are bound to their hospital and treating haematologist. In the group without an earlier trial experience, just under a quarter (21.0%) of patients indicated they would not participate in a trial in case they had to go to another hospital or would participate only in case there was no other treatment available in the current hospital (20.4%). With the certainty of receiving a better treatment, only 15.9% patients would be willing to change hospitals. Another 30.9% patients would go to another hospital for trial participation if their treating physician advised to do so. In the trial population, 14% patients would not consider going to another hospital or would go only if there was no other possible treatment available (27.8%). On the advice of their treating physician, 26% patients would go to another hospital, and 21.7% would go if they were certain about receiving a better treatment.

**Figure 5:**
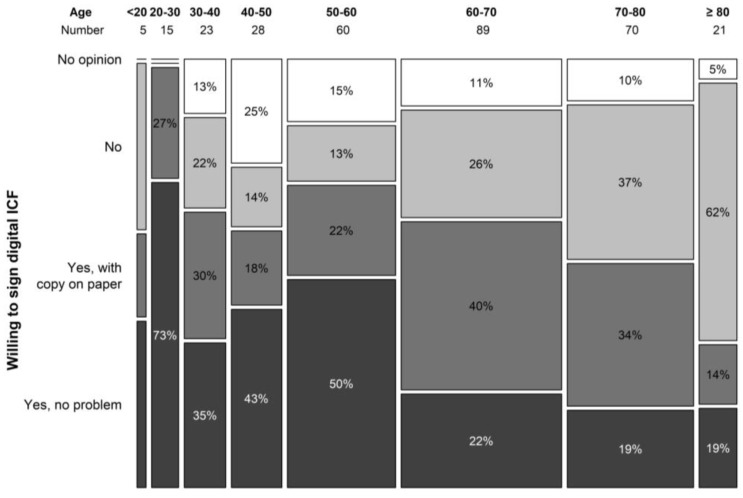
Willingness to sign digital ICF per age category.

The digital ICF procedure was not at all appreciated by 26.1% of the study population that had never participated in a clinical trial. Another 29.9% patients answered they would only agree to a digital ICF as an add-on to a hard-copy procedure. About a third (32.15%) of patients answered they would not have a problem with a digital ICF procedure, and 11.9% did not have an opinion on this matter. In the trial population, we obtained comparable results. The digital ICF procedure was not appreciated by 19.8% patients or only as an add-on to a hard copy by 36.3%. A low proportion of patients had no opinion pertaining to a digital ICF procedure (3.3%), and a larger portion (40.7%) would not have a problem with a digital ICF procedure. Answers differed greatly by age group with a significant difference seen in answers of patients older than 60 years. Overall, 24.5% of patients above 60 years of age indicated the willingness to use a digital ICF procedure vs. 57.0% in the group younger than 60 years (*p* < 0.001) (Figure 5). In terms of financial burden, 12.9% of patients indicated they incurred extra costs due to study participation that were not reimbursed by the study.

### 3.2. Physician Survey

We collected 46 anonymous responses of which 42 were included in the analysis. Four participants ended the survey prematurely as they were not within our targeted survey population. Most responders (47.5%) were younger than 40 years old, 12.5% were between 40 and 50 years old, 27.5% between 50 and 60 years old, and 12.5% older than 60 years old. Most physicians were active in large centres (48.8% university, 48.8% large peripheral, and 2.4% small peripheral) that performed stem cell transplantations (61.9% auto- and allogenous, 21.4% only autologous). A part of physicians, i.e., 16.7%, did not perform stem cell transplantations. Seventeen percent (*n* = 7) did not participate in clinical trials of which half (50%) had never considered it, 25% did not succeed, and 25% were working on it (total responses *n* = 4). Reasons of not participating a clinical trial were as follows (multiple answers were allowed): there was no time, the insufficient support of a data team, the lack of interest in clinical trials, and the lack of knowledge on how to start the process or having a collaboration with a larger centre that performed clinical trials. No one indicated that their current centre was too small for clinical trials, and no indication was made that they have never been contacted for trial participation. No one referred patients on a regular basis for trial participation.

**Figure 6:**
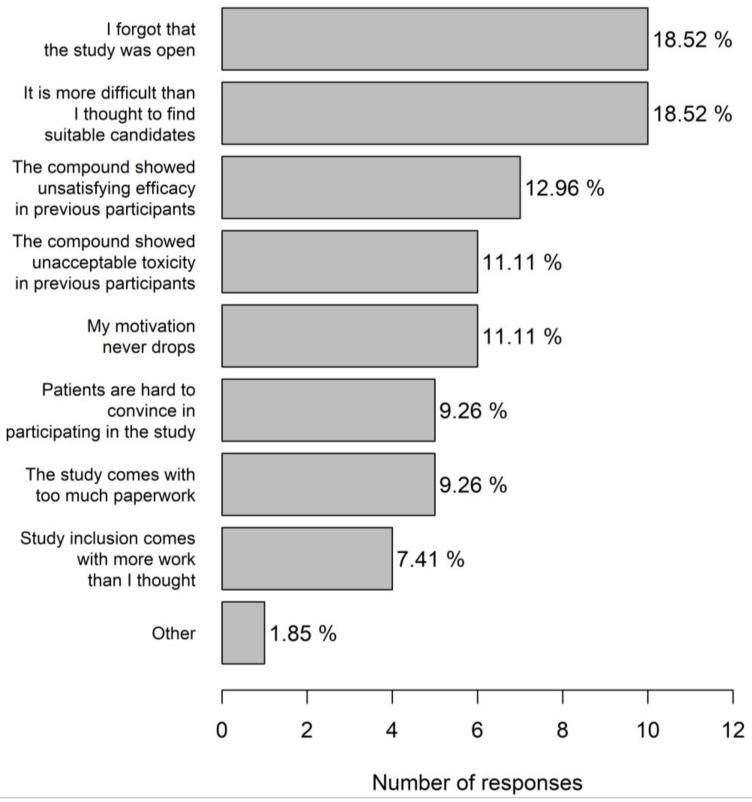
Reasons for decreased physician motivation for trial inclusion.

Most trial centres were active in phase 1–3 studies (70%) including 17.7% centres with a dedicated phase 1 portion. Most physicians (83.3%) considered themselves very motivated for trial participation. The main incentives for trial participation (multiple answers possible) were proven clinical benefit of the molecule (28.4%), scientific interest (27.3%), prestige (20.5%), financial compensation (15.9%), and a good earlier experience with the sponsor (8.0%). A referral to another hospital was made when there was a clear therapeutic benefit of study participation (72.0%) or in case no treatment was available in the current centre (18.8%). Just under ten percent physicians indicated not to refer patients to another centre. A significant portion (21.2%) of physicians had the impression that they probably had difficulties in motivating patients for trial participation, and 22.6% indicated that they did not have a good idea about studies open for inclusion. A decrease in motivation for trial inclusion mainly occurred when physicians forgot that the study was open and when it was found that it was more difficult to find suitable candidates than was first thought (both 18.5%). In 11.1% physicians, the motivation to include patients into a specific trial never decreased (Figure 6). Study emails were completely ignored most of the times by 44.0% physicians.

In nearly all centres (90.9%), a specific study team was available, and regular meetings on clinical trials were organised (94%). The average time between signing a confidential disclosure agreement (CDA) and opening of the study was equally distributed between 8–12 weeks, 12–20 weeks, and 20–30 weeks. Time delay was mostly caused by contract negotiations (78.8%). It is accepted by physicians (31.0%) that currently patients are not ready for a digital ICF (44.8%) or only in combination with a hard-copy procedure.

## 4. Discussion

Due to the lack of enrolment of underserved groups into clinical trials, real-life data are needed and may not correspond to what was concluded from a clinical trial [22]. Accrual limitations originate in the trial design [23]. During site selection and patient inclusion (cultural background, language barriers, logistical aspects, etc.), the eligible population is further thinned out [24]. Even if eligible, patients might be reluctant towards trial participation for different reasons [15]. A new aspect is the digitalisation of different trial procedures, which could possibly form a new barrier or even bias. We explored the knowledge and attitude towards clinical trials and the e-consent procedure in patients with a haematological disease in one survey, and the motivation of haematologists for trial participation in a second survey. A limitation in the organization of these surveys is the inability to estimate the participation rates. Patients can see the survey as a study, thereby under-representing patients who are more aversive towards clinical trials. The survey was only available in Dutch since this is by far the most frequent language used amongst our patients; nevertheless, patients who are not familiar with the language are not represented here. The different hospitals in which patients were invited for participation are mostly perceived as city hospitals and could attract a different population compared to more rural hospitals. We did not collect information on health literacy, which previously has shown to have an impact on trial invitations but not on enrolment [25]. The patient survey had 420 responders, of which 100 had experience with trial participation. In the physician survey, we included 42 participants with a chance that the invitation by email was missed by a greater number of physicians. We mainly reached younger haematologists working in university centres and large non-university centres. 

Older patients should not be excluded from trial invitation [26]. With age, the disparity in inclusion between clinical trials and real life is commonly noted [27]. Although our results show that the motivation for trial participation seems to decline with advanced age, this was borderline non-significant. Most of our patients (*n* = 239) are 60 years or older. In this age group, the use of an electronic ICF is not the preferred method. An observation that is shared by most haematologists. This finding should prevent us from rushing into digitalizing this aspect of clinical trials. The lack of understanding about clinical trials is a known barrier, and nowadays, most information is digitalized. This motivated us to develop, print, and distribute a hard copy information brochure on clinical trials. The development of the brochure was conducted in collaboration with the patient committee of the Belgian Haematological Society. The brochure is available for free in French and Dutch in nearly every hospital in the country. We hereby aim to overcome the information barrier in the elderly population. A digital version of the brochure can be found on the website of the Belgian Haematology Society.

In total, 12.8% (*n* = 53) of the patients reported to be familiar with the general concepts of clinical trials. The knowledge is in line with a previously described Italian population, in which 19% of patients was found to have a good understanding of clinical research [28]. Specifically in our population, this finding is notable considering that 23.9% (*n* = 100) previously participated in a clinical trial. This expresses a high level of confidence in clinical trials that was confirmed by the general attitude towards clinical studies, safety concerns, and the willingness to participate in a trial when offered. Patients had more knowledge on the conduction of clinical trials had a significant higher willingness to participate in a trial. A similar observation was made for patients with a better patient–doctor relationship that resulted in a higher confidence and significantly more willingness to be enrolled. In our cohort, we found 10% participants were aware of clinical trial concepts but not willing to participate. Six patients had refused trial participation in the past. In 50% of these patients (*n* = 3), this was due to a lack of faith in pharmaceutical companies. It is a reminder that continued efforts are needed since unethical trial conduct is not just a thing of the past [29].

Patients that previously participated in a clinical trial were satisfied with the information that was provided in the ICF, and its current format is well accepted. Reporting of trial results to the patients is preferred by the majority of participants and should be a standard procedure, with a possible negative checkbox for those who do not wish to receive the results.

Most patients are not enthusiastic about the idea of referral to another hospital for trial participation. Haematologists would refer a patient to another centre in case there was a clear patient benefit. However, a significant portion of physicians would exhaust every other therapeutic option before referring to their patients to another centre or would not refer at all. Prestige and financial remunerations as motivators for trial inclusion and could be the basis of this attitude. Since many patients and haematologist are comfortable with the idea of minimal referral, augmenting referrals for clinical trials is a challenge. This behaviour might not be perceived as unethical towards a single patient; however, it decreases the likeliness of success in being enrolled later. With respect to the community as whole, it hinders medical progress. Despite the difficulty in referring for a trial, referral is more likely with a strong patient–physician relationship. 

Haematologists who are not active in clinical trials perceive practical aspects as the main barrier and not missing invitations. Sponsors are responsible for the e-mail traffic they generate. In the current setup, nearly half of the haematologists indicated to completely ignore study e-mails most of the time. This reflects a failure of the current system of notifications.

Administrative procedures are known to be time-consuming [30]. Despite the organisational structures that are outlined by a dedicated study team, there is a strong delay between signing CDA and opening of the study that is mostly attributed to contract negotiations. Setting up a national trial finance committee to install fixed charges and remunerations that apply to each centre could be a possible step forward. 

Apart from practical aspects such as a forgotten study or no suitable candidates, a decreased motivation is mainly based on a previous negative experience with a product under investigation. Although this attitude is scientifically incorrect, this implies that the patient always comes first.

## 5. Conclusions

This study confirms the high willingness of patients, irrespective of their age, and haematologists for trial participation in Belgium. Haematologists have a key role in augmenting trial inclusions. A strong patient–physician relationship leads to a higher willingness to participate in a trial, a more open communication, and a higher willingness to provide referral. Development of central agreements on clinical trials is necessary to bolster current structures that are the major cause of delay in starting new studies. Rushing into digitalizing clinical trial procedures should be avoided since the implementation of an e-consent is not preferred by most haematological patients over 60 years.

## Data Availability

The dataset used and analysed during the current study is available from the corresponding author on reasonable request.

## Supplementary Materials

The following supporting information can be downloaded at: https://www.mdpi.com/article/10.3390/clinpract13060133/s1, File S1: Patient survey on clinical trials (translated from Dutch); File S2: Physician survey on clinical trials.
