# Supplementary material for: Combined Results of Two Cross-Sectional Surveys on the Participation in Clinical Trials and the e-Consent Procedure in the Landscape of Haematology
Source: Clin Pract. 2023 Nov 23;13(6):1520–31. doi: 10.3390/clinpract13060133 (PMC10742482; doi:10.3390/clinpract13060133)
Supplement: Supplementary file 1 [file clinpract-13-00133-s001.zip › File S1---Revised Supplementary S1 Patient-Survey on clinical trials (translated from Dutch).pdf]

Supplementary S1: Patient-Survey on clinical trials (translated from Dutch)

What is your gender?	%	n
- Female	53.81%	219
- Male	46.19%	180
- Gender X		0
- I prefer not say		0
	Total	407

What is your current age?		
- Younger than 20 years old	1.9%	8
- Between 20 and 30 years old	4.76%	20
- Between 30 and 40 years old	8.81%	37
- Between 40 and 50 years old	7.86%	33
- Between 50 and 60 years old	19.76%	83
- Between 60 and 70 years old	29.29%	123
- Between 70 and 80 years old	20.95%	88
- Older than 80 years old	6.67%	28
	Total	420

Do you regularly use the internet?		
- Yes, I use internet on a daily basis	77.29%	320
- Yes, I use internet at least once per week	9.42%	39
- No, I use internet less than once per week	3.14%	13
- No, I rarely or never use the internet	10.14%	42
	Total	414

How well are you informed on the general concepts of clinical trials?		
- I am very well informed	12.8%	53
- I have limited knowledge on clinical trials	36.44%	151
- I am not really informed on clinical trials	30.92%	128
- I am totally not informed on clinical trials	19.81%	82
	Total	414

How would you describe your general attitude towards clinical trials?		
- Very positive	37.92%	157
- Rather positive	50,00%	207
- Rather negative	16.63%	44
- Very negative	1.45%	6
	Total	414

How safe do you think clinical trials are?		
- Very safe	21.26%	88

-	Rather safe	65.94%	273
-	Not really safe	11.11%	46
-	Not safe at all	1.69%	7
		Total	414

What do you think, can be possible risks when participating in a clinical trial? (Multiple answers possible)

-	Possible side-effects	40.84%	292
-	The possibility to receive a non-active treatment (placebo)	26.01%	186
-	Publicly disclose my medical file	5.13%	36
-	Risks about my general health	20.14%	144
-	I don't really think there are risks involved	7.97%	57
		Total	715

What do you think can be possible advantages that clinical trials bring for society? (Multiple answers possible)

-	Contribution to scientifical progress	43.75%	343
-	Amelioration of the health care system	25.77%	202
-	Save lives of patients	28.57%	224
-	I don't think there are any advantages	1.91%	15
		Total	784

What do you think can be personal advantages in case you would participate in a clinical trial? (Multiple answers possible)

-	It helps in treating my disease	40.18%	307
-	I can get money in case of participation	3.4%	26
-	I will receive free medication	9.29%	71
-	I will get more attention from my treating physician	9.95%	76
-	It helps for my family to understand the disease	7.46%	57
-	It brings satisfaction from helping others by participation	24.61%	188
-	I don't think there are personal advantages	5.1%	39
		Total	764

In case you would be invited for participation in a clinical trial, to what extent you be willing to participate?

-	Very willing to participate	28.09%	116
-	Rather willing to participate	39.71%	164
-	Not really willing to participate	15.98%	66%
-	Not at all willing to participate	1.6%	19
-	I don't know	11.62%	48
		Total	413

In case you would consider participation in a clinical trial, where would you obtain information? (Multiple answers possible)

-	With my general practitioner	26.48%	197
-	With my treating haematologist	39.92%	297
-	Other healthcare workers (nurses, psychologist, physiotherapist…)	9.41%	70

- Friends and family	4.44%	33
- Patient groups	4.03%	30
- Internet	12.5%	93
- Social media (Facebook, twitter, YouTube…)	1.48%	11
- I don’t know	1.75%	13
	Total	744

Did you participate in a clinical trial in the past or are you currently participating in a clinical trial?

- Yes	23.81%	100
- No	76.91%	320
	Total	420

Did you refuse trial participation in the past?

- Yes	1.45%	6
- No	98.55%	413
	Total	419

What was the main reason for refusal?

- I was not convinced of the proposed treatment	0%	0
- I was given too little information	16.67%	1
- My treating physician had insufficient answers to my questions	16.67%	1
- Trial participation came with more frequent hospital visits	16.67%	1
- I have no trust in the pharmaceutical industry	50,00%	3
- I was afraid of possible side effects	0%	0
- I needed to go to another hospital	0%	0
- None of the above	0%	0
	Total	6

Would you be willing to go to another hospital for trial participation in case your doctor would refer you?

- No, in that case I would not participate	21.02%	66
- Yes, but only in case there is no other treatment in my current hospital	20.38%	64
- Yes, in case I could have a better treatment compared to my current hospital	15.92%	50
- Yes, in case my doctor would advise me to do so	30.89%	97
- Yes, I search actively for open clinical trials	0.56%	3
- I don’t know	10.83%	34
	Total	314

In case you would give your consent for participation in a clinical trial, would you agree to place a digital signature (on a computer) and receive the information digitally (on computer)?

- No, I prefer pen and paper	26.05%	81	19.78% *	18*
- Yes, but I prefer to have all information and signed documents on paper as well	29.9%	93	36.26% *	33*
- Yes, I would have no problem with this	32.15%	100	40.66% *	37*
- I don’t have an opinion on this	11.9%	37	3.30% *	3*
	Total	311	Total*	91*

*population with previous or current trial participation

In case you would participate in a clinical trial, would you like to know the results of the trial once they are known?

-	No, I have no need to know this	6.69%	21
-	No, unless this would be important for my future treatment	5.73%	18
-	Maybe yes, I would like to state this in advance whether or not I would like the results	7.64%	24
-	Yes, I would like to know the results from the moment they are known	79.94%	251
		Total	314

Do you know someone who has participated in the past or is currently participating a clinical trial (regardless of the disease)?

-	No, I don't know anyone who has ever participated	62.74%	197
-	Yes, but not personally	14.65%	46
-	Yes, I do know people who participated in a clinical trial	22.61%	71
		Total	314

How would you score your relation with your treating haematologist with a maximum of 10 points? (This questionnaire is fully anonymous; your treating haematologist will not know the answers).

-	1	0%	0	1.10%*	1*
-	2	0%	0	1.10%*	1*
-	3	1.29%	4	1.10%*	1*
-	4	0.96%	3	0%*	0*
-	5	3.54%	11	1.10%*	1*
-	6	4.18%	13	2.20%*	2*
-	7	19.94%	62	2.20%*	2*
-	8	26.37%	82	30.77%*	28*
-	9	23.97%	74	23.08%*	21
-	10	19.94%	62	37.36%*	34*
		Total	311	Total*	91*

*population with previous or current trial participation

Specifically for patients that responded 'yes' to the question: 'Did you participate in a clinical trial in the past or are you currently participating in a clinical trial?'

How do you feel about the amount of information you received on the clinical trial you participated in or are currently participating?

- The information was too detailed, I don't need to know so many details	6.38%	6
- The amount of information was correct	73.4%	69
- I would have liked more information than was given	13.83%	13
- I don't remember	6.38%	6
	Total	94

Could you comprehend all information that was given?

- I comprehended everything	58.06%	54
- I comprehended most of it, some parts I could not comprehend	32.26%	30
- I comprehended very little of the information that was offered	4.3%	4
- I cannot remember any information was given	5.38%	5
	Total	93

Did you have enough time for questions?

- Yes, there was sufficient time for questions	84.95%	79
- No, there was not enough time for questions	10.75%	10
- I don't remember	4.3%	4
	Total	93

Where you satisfied with the answers that were given to your questions?

- Yes, completely	55.91%	52
- Yes, most of the time	36.56%	34
- No, I was not satisfied with the answers	3.23%	3
- I did not have questions	2.15%	2
- I cannot remember	2.15%	2
	Total	93

The informed consent form (ICF) is built up in accordance with scientific guidelines. I think that the current format of the ICF is …. (Multiple answers possible)

- Correct, the current form does not influence my decision to participate	54.03%	53
- Too scientific, which makes it difficult to understand the information	11.22%	11
- Not invitational to read, I would prefer more colour and graphics	11.22%	11
- I don't have an opinion on this	23.47%	23
	Total	98

I incur additional costs by participating in a clinical trial.

- Yes	12.9%	12
- No	68.02%	64

- Yes, but they are reimbursed by the trial sponsor	13.98%	13
	Total	93

During the study, I have had the sensation of being a test-object.

- Yes	5.31%	5
- No	76.34%	71
- Yes, but I could speak about this with the study team or treating physician	13.98%	13
- I cannot remember	4.30%	4
	Total	93

I would advise other patients to participate in a clinical trial

- Yes	75.27%	70
- Yes, but only to younger patients	5.38%	5
- Yes, but only to elderly patients	4.3%	4
- No, I would not advise this	2.15%	2
- I don’t have an opinion on this	12.9%	12
	Total	93

I would participate in another clinical trial, in case I was invited to do so.

- Yes	51.61%	48
- Yes, in case I have a chance on a better treatment	40.86%	38
- Yes, but only in case there is no other treatment available	5.38%	5
- No	2.15%	2
	Total	93
