# Supplementary material for: Combined Results of Two Cross-Sectional Surveys on the Participation in Clinical Trials and the e-Consent Procedure in the Landscape of Haematology
Source: Clin Pract. 2023 Nov 23;13(6):1520–31. doi: 10.3390/clinpract13060133 (PMC10742482; doi:10.3390/clinpract13060133)
Supplement: Supplementary file 1 [file clinpract-13-00133-s001.zip › File S2---Revised Supplementary S2 Physician-Survey on clinical trials.pdf]

Supplementary S2: Physician-Survey on clinical trials

I currently work as a

- Clinical haematologist	86.96%	40
- Oncologist	8.70%	4
- Haematologist/oncologist in training	4.35%	2
- None of the above // end of survey		
Total	Total	46

My current age is

- <40 years old	47.5%	19
- Between 40-50 years old	12.5%	5
- Between 50-60 years old	27.5%	11
- >60 years old	12.5%	5
	Total	40

The hospital where I perform my main activity is a …

- University Hospital	48.78%	20
- Large peripheral hospital (>400 beds for hospitalization)	48.78%	20
- Small peripheral hospital (<400 beds for hospitalization)	2.44%	1
	Total	41

Are stem-cell transplantations performed in your centre?

- Yes, allogeneic and autologous	61.9%	26
- Yes, only autologous	21.43%	9
- No	16.67%	7
	Total	42

Are there interventional haematological studies running in your centre?

- Yes, some phase 1, mostly phase 2 and 3	53.66%	22
- Yes, phase 1,2 and 3 with a dedicated phase 1 portion	4.88%	2
- Yes, some phase 1, mostly phase 2 and 3	7.32%	3
- Yes, phase 3 only	17.07%	7
- No	17.07%	7
	Total	41

IF NO to last question

Did you ever consider attracting clinical trials to your centre?

▪ Yes, I'm working on it	25%	1
▪ Yes, but I did not succeed	25%	1
▪ No, I have not considered this	50%	2
	Total	4

What are the main reasons why there are no clinical trials in your centre? (multiple answers possible)

▪ I don't have time for this	16.67%	1
▪ There is insufficient support of a data-team	16.67%	1
▪ I am not interested in participating in clinical trials	16.67%	1
▪ I would not know how to start this process	16.67%	1
▪ There is a collaboration with another centre that performs clinical trials	33.33%	2
▪ The centre where I work is too small for interventional trials	0%	0
▪ I have never been contacted for participation	0%	0
	Total	6

Did you refer a patient to another centre to participate in a clinical trial during the last year?

▪ Yes, I refer patients regularly for clinical trial participation	0%	0
▪ Yes, there was no therapeutic alternative in my centre	100%	4
▪ Yes, but I would not do this anymore	0%	0
▪ No, there was no medical need for patient referral	0%	0
▪ No, I would not do this	0%	0
	Total	4

During the last year, did you refer patients to another centre for CAR-T cell infusion?

This question was not retained for the analysis of this manuscript

What are/could be the 3 main criteria in the choice of referral to a CAR-T cell infusion centre?

This question was not retained for the analysis of this manuscript

I consider myself motivated to include patients in clinical trials

- Yes, very motivated	83.3%	15
- Yes, but maybe not enough	11.1%	2
- Yes, but only for certain studies	5.56%	1
- No, not so much	0%	0
	Total	18

Does your department have a specific study team/data nurses?

- Yes	90.91%	30
- No	9.09%	3
	Total	33

Are there regular meetings dedicated to clinical trials?

- Yes	94.12%	32
- No	5.88%	2

		Total	34

What can be incentives to participate in a clinical trial? (multiple answers possible)

-	Financial compensation	15.91%	14
-	Prestige (e.g. Only centre in the area that offers the study)	20.45%	18
-	Proven clinical benefit of the molecule	28.41%	25
-	Scientific interest	27.27%	24
-	Good experience with the sponsor	7.95%	7
		Total	88

Does your centre participate in CAR-T cell trials?

This question was not retained for the analysis of this manuscript

What is the average time between signing the CDA (confidential disclosure agreement) and opening of a study?

-	<8 weeks	0%	0
-	8-12 weeks	36.36%	12
-	12-20 weeks	30.30%	10
-	20-30 weeks	30.30%	10
-	>30 weeks	3.03%	1
		Total	33

This is mainly due to…

-	Study initiation by the sponsor of the study	12.12%	4
-	Contract negotiations	78.79%	26
-	The ethical committee	3.03%	1
-	None of the above	6.06%	2
		Total	33

Are you satisfied with a print-out of power-point slides at study initiation?

-	Yes, this does not bother me	48.48%	16
-	No, I would prefer the same information in some pages	27.27%	9
-	I have no opinion on this	24.24%	8
		Total	33

During the last year, did you refer a patient to another centre to participate in a clinical trial?

-	Yes	62.5%	20
-	No	37.5%	12
		Total	32

When would you refer a patient to another centre?

-	Only if there is no treatment available in my centre	18.75%	6
-	When there is a clear therapeutic benefit of study participation	71.88%	23
-	I don’t refer patients to another centre	9.38%	3
		Total	32

Do you have any personal financial benefit of including patients in clinical trials?

-	Yes	25%	8
-	No	71.8%	23
-	I don’t know	3.13%	1
		Total	32

I have the impression that I have difficulties in motivating patients to participate in clinical trials.

-	Definitely yes	0%	0
-	Probably yes	21.21%	7
-	Probably not	51.52%	17
-	Definitely not	27.27%	9
		Total	33

I don’t have a good idea about the studies that are open for inclusion in my centre.

-	Yes, this is correct	22.58%	7
-	No, I know exactly what studies are currently open	64.52%	20
-	No, only some studies because they carry my interest	12.9%	4
		Total	31

When my motivation to include patients in study decreases, this is due to (multiple answers possible)

-	I forgot the study was open	18.52%	10
-	Patients are hard to convince in participating in the study	9.26%	5
-	It is more difficult than I thought to find suitable candidates	18.52%	10
-	Study inclusion comes with more work than I thought	7.41%	4
-	The compound showed unsatisfying efficacy in previous participants	12.96%	7
-	The compound showed unacceptable toxicity in previous participants	11.11%	6
-	The study comes with too much paperwork	9.26%	5
-	My motivation never drops	1.85%	1
		Total	48

Most of the time, I completely ignore study emails

-	Yes	43.73%	14
-	No	56.25%	18
		Total	32

Inclusion targets are mostly reached when a trial runs in your centre.

-	Yes, always	3.23%	1
-	Yes, most of the times	80.65%	25
-	No, most of the times not	12.9%	4
-	No, never	3.23%	1
		Total	31

After study-closure, an overview of the financial balance is presented to me or someone in my centre in way that there is a clear view on the costs made, the resources received and the resources that still need to be invoiced.

-	Yes	6.9%	2

-	Sometimes	31.03%	9
-	No	31.03%	9
-	I have no idea	31.03%	9
		Total	29

In the past, I have postponed a treatment (medically justified) in order to have a patient participate in a clinical trial.

-	Yes	62.07%	18
-	No, but I would	27.59%	8
-	No and I would not do this	10.34%	3
		Total	29

I think patients are ready to use an electronic ICF and digital signature, without a printed version.

-	Yes	24.14%	7
-	Yes, but with a print-out they can take home	31.03%	9
-	No	44.83%	13
		Total	29

Patients should be able to indicate in the ICF if they wish to be informed about the study-results once they are known.

-	Yes	89.66%	26
-	No	3.45%	1
-	I have no opinion on this	6.90%	2
		Total	29
